# Fabrication, Acoustic Characterization and Phase Reference-Based Calibration Method for a Single-Sided Multi-Channel Ultrasonic Actuator

**DOI:** 10.3390/mi13122182

**Published:** 2022-12-09

**Authors:** Hiep Xuan Cao, Daewon Jung, Han-Sol Lee, Van Du Nguyen, Eunpyo Choi, Chang-Sei Kim, Jong-Oh Park, Byungjeon Kang

**Affiliations:** 1 School of Mechanical Engineering, Chonnam National University, Gwangju 61186, Republic of Korea; 2 Korea Institute of Medical Microrobotics, Gwangju 61011, Republic of Korea; 3 College of AI Convergence, Chonnam National University, Gwangju 61186, Republic of Korea; 4 Graduate School of Data Science, Chonnam National University, Gwangju 61186, Republic of Korea

**Keywords:** ultrasonic actuators, phase calibration, twin trap

## Abstract

The ultrasonic actuator can be used in medical applications because it is label-free, biocompatible, and has a demonstrated history of safe operation. Therefore, there is an increasing interest in using an ultrasonic actuator in the non-contact manipulation of micromachines in various materials and sizes for therapeutic applications. This research aims to design, fabricate, and characterize a single-sided transducer array with 56 channels operating at 500 kHz, which provide benefits in the penetration of tissue. The fabricated transducer is calibrated using a phase reference calibration method to reduce position misalignment and phase discrepancies caused by acoustic interaction. The acoustic fields generated by the transducer array are measured in a 300 mm × 300 mm × 300 mm container filled with de-ionized water. A hydrophone is used to measure the far field in each transducer array element, and the 3D holographic pattern is analyzed based on the scanned acoustic pressure fields. Next, the phase reference calibration is applied to each transducer in the ultrasonic actuator. As a result, the homogeneity of the acoustic pressure fields surrounding the foci area is improved, and the maximum pressure is also increased in the twin trap. Finally, we demonstrate the capability to trap and manipulate micromachines with acoustic power by generating a twin trap using both optical camera and ultrasound imaging systems in a water medium. This work not only provides a comprehensive study on acoustic actuators but also inspires the next generation to use acoustics in medical applications.

## 1. Introduction

Non-contact manipulation of microparticles has received a lot of attention in biomedical applications such as drug carrier delivery, cell manipulation, and sorting. However, the targeted particles must be controlled for these techniques to achieve precise motions, and acoustic tweezers are recommended [1,2,3,4,5,6]. In addition, acoustic sources can produce dragging or repelling points, which can collect particles of various sizes and types in specific locations [7,8,9,10,11]. Several ultrasound-based approaches have been demonstrated in previous studies due to these advantages.

One of the most common approaches is to generate acoustic pressure fields using a standing wave [12,13,14]. This wave applies a stable force to the targeted object, but the field can only be generated by parallel ultrasound transducer arrays reflecting the emitted wave [15,16,17]. This structure is complex and unsuitable for some application scenarios, particularly when the array can only be used on one side of the targeted trapping region. On the other hand, a traveling wave generates the pressure field without a reflector, making the single-sided array suitable for use in vivo [18,19,20,21,22,23]. A single acoustic beam is produced when multiple traveling waves overlap at the same point. The point can also be relocated by phase modulation of each transducer to manipulate targeted particles into specific positions [18,24,25,26]. Marzo et al. presented 3D holographic patterns for trapping (i.e., twin, bottle, and vortex traps) based on this approach [22,27,28]. All these can trap and manipulate some targeted particles with any hemispherical arrangement of multiple transducers, but the twin trap is considered a suitable pattern for providing two extra rotational motions for the acoustic tweezer [29].

**Figure 1:**
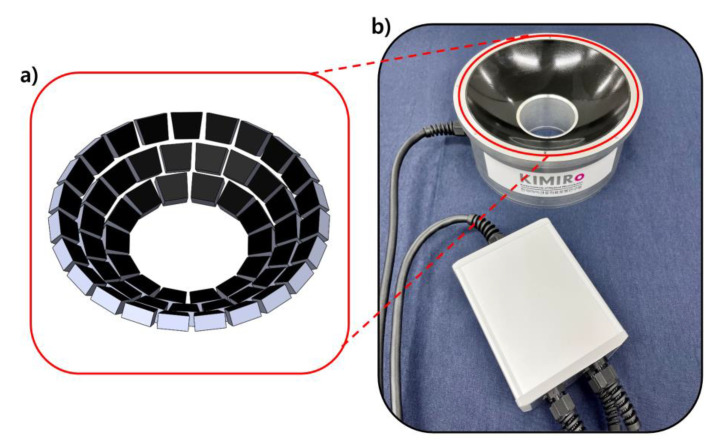
Overview of 56-channel model: (**a**) Arrangement of 56 transducers; (**b**) Photograph of the model: (Top) 56-ultrasound array body, (Bottom) Relay box for distribution of 56 channels.

The concept of a hemispherical arrangement of ultrasound transducers has been applied for many applications, including ablation of deep tumors and neurological diseases [30,31,32,33,34]. Instead of these conventional applications, we focus on the manipulation of microparticles for targeted drug delivery as our future application. In the conceptual stage of our array model, several design criteria are set for the targeted purpose. The optimal focal distance and resonance frequency are chosen for the surgical environment of the targeted organs, and each phase value of transducers can also be modulated to achieve desired motions for various medical applications. Based on these criteria, this research aims to investigate a design, characterization, and phase calibration method for a 500 kHz, 56-element transducer array. First, we describe how to design and build the 56-element transducer array that operates at 500 kHz, which is beneficial for tissue penetration. The 56 transducers are arranged as shown in Figure 1a inside our fabricated array model (Figure 1b). Each transducer is trapezoidal to increase the filling factor of the array’s active area. The array’s holographic pattern is then checked as the simulated acoustic pressure field. Normal single beam and twin trap simulations are performed in COMSOL Multiphysics using the designed model. The simulation results show the expected patterns. Next, we scanned the acoustic field using a hydrophone and compared it to the simulation results. Finally, phase calibration is performed to improve the array’s performance. The hydrophone, located at the array’s focal point, can measure phase differences, allowing these values to be matched with a reference phase. Using this approach, our system will be improved for trapping and manipulating micromachines in future work.

In this paper, the design of the system is presented in Section 2.1, whereas Section 2.2 provides the acoustic feature of the array. The calibration method is presented in Section 2.3, Section 3 present the result and discussion, and the conclusion is presented Section 4.

## 2. Materials and Methods

### 2.1. Design and Fabrication of Acoustic Actuator

**Figure 2:**
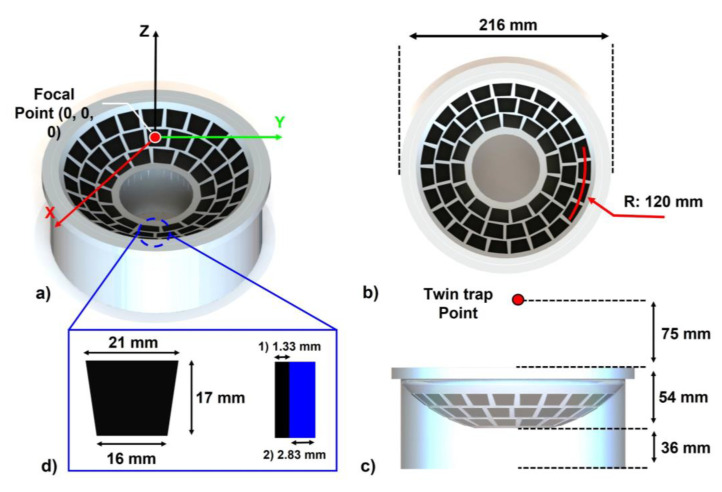
Single-side UTs array geometry design: (**a**) isometric view; (**b**) top view; (**c**) lateral view; and (**d**) size of the single element and thickness of matching layer and PZT layer.

The multi-element transducer array is designed to meet our medical application requirements. The physical focal point in the array is determined by the radius of curvature. If the point is close to the array’s surface, the workspace would be insufficient for some medical applications. Constantly, a high-focal distance from the surface can provide enough space while causing a pressure drop at that point. The resonance frequency is related to the penetration depth and the targeted particle’s size. When the frequency is increased, the acoustic wave exhibits a shorter wavelength, allowing for the manipulation of smaller particles in the array. For the experimental condition, the array should be built as an immersion type. The acoustic impedance of water (1480 × 10^3^ kg/s·m^2^) is significantly similar to that of human tissue (1530 × 10^3^ kg/s·m^2^) [35,36,37], so it is used as a medium in various medical applications [38,39,40,41]. The array contains 56 trapezoidal transducers arranged in a hemispherical pattern to match the focal points of the transducers, as shown in Figure 2. This transducer is composed of two layers, as in Figure 2d. The impedance matching layer was designed to meet the acoustic impedance of water with a thickness of 1.33 mm and a PZT layer of 2.83 mm to generate desired acoustic waves under 500 kHz. The transducer shape is chosen to maximize the filling factor in each line. Table 1 shows the specifications of the proposed single-sided UTs, and these chosen values are decided by following the design requirements. Firstly, the system’s resonance frequency was decided based on the aspects of ‘Permeability’ and ‘Interference with the imaging probe’. When compared with the over-a-megahertz system, the under-a-megahertz system has a longer wavelength that can more easily penetrate the superficial tissue. Furthermore, we plan to combine our system with an ultrasound imaging system in the future, and most imaging systems use a megahertz range. Thus, the under-a-megahertz range can avoid interference with the imaging system. Based on these merits, we choose 500 kHz as the resonance frequency. Next, the proposed ultrasound array system will be applied in various medical applications for the liver, and the average depth from the skin to the liver is 25 mm [42]. In consideration of the unusual situation, we fix a depth of 3 times the average depth (75 mm) as the focal distance. Finally, the number of transducers is chosen with the following considerations: We used commercial PZT (Japan Probe, Yokohama, Japan), which has an area of 314.5 mm^2^, and this PZT should be arranged with a minimum gap of 2 mm to reduce sidelobes. Using these two design criteria, we can decide the optimal number of transducers that are arranged in a hemispherical shape. However, we also have to consider the space for a center hole (70 mm) that can be used for the imaging probe, and a focal distance of 75 mm in the array. In order to satisfy all these considerations, 56 is chosen as the number of transducers.

We use custom immersible transducers, and the acoustic characteristics of the transducers can provide specific data for the simulation model in COMSOL. Based on this model, the optimal radius of curvature is decided as 120 mm, which can keep a stable acoustic pressure at the focal point. To maximize the workspace of the array, the proper number of transducers should be arranged with a small gap that starts from the center of the array. In the acoustic field, sidelobes are also generated in the area and these lobes give a negative effect on the uniformity of the field. According to Rosnitskiy PB et al. [43], the lobe can be suppressed by densely arranged transducers. The ideal gap between each transducer is less than half of the wavelength (Wavelength λ = Speed of Sound C/Frequency f). We use 500 kHz in water (1500 m/s), thus 1.5 mm is the ideal value, but due to the limitations of transducer fabrication, each transducer was arranged with a 2 mm gap. Through these design criteria, the array comprised 56 transducers with a workspace height of 75 mm. We built our customized signal amplifier in a previous study to drive 56 transducers in the array independently [44]. The amplifier can amplify a signal in the 10–100 Vpp range with a maximal current of 0.5 A in each channel. Based on that system, we added 32 more channels to control independent square wave signals. These signals included the power source’s amplitude and phase data.

### 2.2. Acoustical Characterization

We describe the acoustical characterization of the acoustic actuator, designed and fabricated with 56 identical transducers, in this section. The UT was modeled numerically in the finite element analysis software COMSOL Multiphysics 5.3 (COMSOL lnc., Burlington, MA, USA) using a planar transducer with 56 elements. The parameters were defined under water conditions. The operating frequency was f = 500 KHz, the density was ρ = 1000 kg/m^3^, and the speed of sound in water is 1500 m/s. The numerical acoustic value in the far field can be calculated for ease of simulation using a single-frequency piston with acceleration control. The equation gives the complex acoustic pressure *P* at point *r (x, y, z)* as:

(1) $$P_{i}(r)=P_{0}A\frac{2J_{1}(\mathrm{kasin}\theta )}{d\times ka\sin\theta }e^{i\left(\varphi +kd\right)}$$

*r (x, y, z)* denotes the distance between the point r at the far field to the center of the *i*th UT. P_0_ denotes a constant defined as the UT’s *i*th output efficiency. *A* denotes the peak-to-peak amplitude of the excitation signal to the *i*th UT. *J_1_* denotes a first-order Bessel function of the first kind. In this paper, we assume that the *i*th UT has an area S and is simulated as a circular piston with a radius *a*. The term *1*/*d* is a correction factor for divergence, where *d* is the free-space propagation distance. The wavenumber is defined as $k=\frac{2\pi }{\lambda }$ with the wavelength *λ*. The phase of the excitation signal in the piston source is denoted as *φ* in radians.

We conducted three measurements to determine the acoustic characterization of UA: (1) element-by-element in far-field measurements, (2) holography measurements, and (3) a 3D scan to determine the pressure uniformity of the twin beam shape.

All three measurements were taken in a 300 mm × 300 mm × 300 mm container filled with de-ionized water. The designed UA was immersed in the water container and connected to the custom high-voltage, high-frequency amplifier via coax cable RG213 50 Ω. All measurements were made with a needle hydrophone HNC 0400 (Onda Corporation, Sunnyvale, CA, USA) and calibration data at 500 kHz. According to the manufacturer’s calibration, the HNC 0400 hydrophone sensitivity at 500 kHz was 0.791 V/MPa. The hydrophone was mounted on the three-dimensional (3D) scan system with a displacement step of 20 μm. We used three types of power units to supply power to the amplifier. Two P3030 units (Advantek, Hayward, CA, USA) with a limited current of 5 A and an accuracy of ±0.1 V powered the regulated 5 V and 3.3 V DCs. To supply bipolar power from ±5 V to ±50 V, an Exso K633A unit (Exso, Busan, Republic of Korea) with a limited current of 5 A and an accuracy of ±0.1 V was used.

#### 2.2.1. Element-by-Element in Far-Field Measurements

Theoretically, the focal distance at which a single transducer generates maximum pressure can be calculated as follows:

(2) $$F_{i}=\;\frac{{\left(2a\right)}^{2}}{4\lambda }$$

This study designed the transducer array to control the far-field region. Therefore, the desired control trap point must be generated at a distance *Z* > *F_i_*. The hydrophone was positioned at the array’s focal point to measure the pressure of a single transducer. Each transducer is driven independently by the amplifier at 500 kHz and a continuous square wave signal with the same phase delay value of 0°. WaveRunner 8000 high-definition oscilloscopes captured the waveform from the hydrophone pre-amplifier (Teledyne LeCroy, Chestnut Ridge, NY, USA). The amplitude and phase were calculated using a discrete Fourier transform (DFT) function and a band-pass filter on the oscilloscopes. As a result, the far-field region yields an effective surface pressure amplitude *P*_0_ and an acoustic power *W_p_* [45,46]:

(3) $$P_{0}=P_{z}\frac{Z\lambda }{\pi a^{2}}$$

(4) $$W_{p}=P^{2}{}_{z}\frac{Z^{2}\lambda^{2}}{2\rho c\pi a^{2}}$$

*P_z_* denotes the pressure amplitude measured at the desired focus point, *Z* denotes the designed focus distance of the transducer array, *a* denotes the radius of each transducer in the array, *W_p_* denotes the acoustic power of the single transducer, *c* denotes the speed of sound in water (1500 m/s), *ρ* denotes the density of water (1000 kg/m^3^), and *λ* denotes the acoustic wavelength (3 mm). In the final step, the acoustic pressure and total power of a single element in the array were then calculated.

#### 2.2.2. Holography Measurements

**Figure 3:**
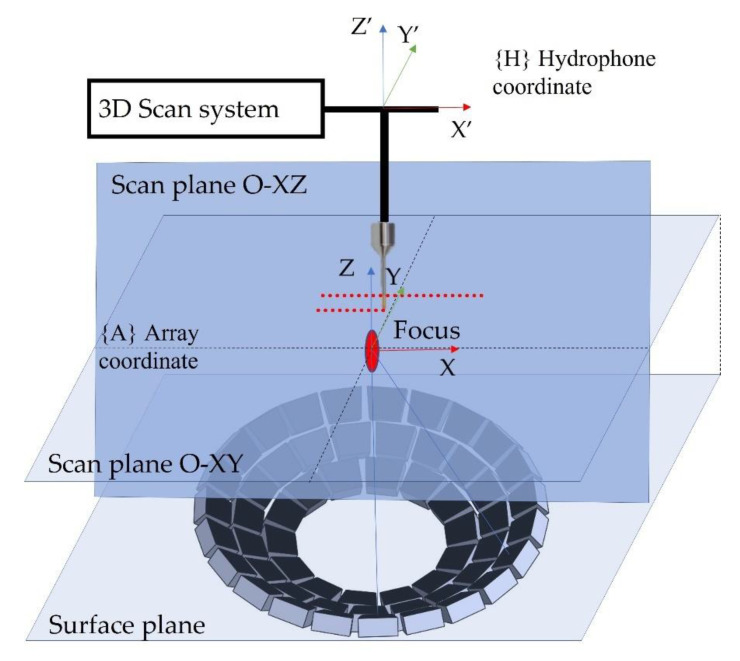
Holography measurement setup for the UA.

The holography measurements begin with an O-XY plane scan of the UA. The plane scan was performed in the center of a 10 × 10 mm^2^ region with a 200 μm displacement step 75 mm from the UA surface plane. All transducers were driven at 500 kHz, 20 Vpp, and 0° phase delay. There are two coordinate systems to consider when measuring pressure in the O-XY plane: the array coordinate system {A}, which is aligned with the UA, and the hydrophone coordinate system {H}, which is aligned with the hydrophone position. The misalignment of the two coordinate systems impacts the pressure measurement. This misalignment could be corrected by utilizing two scan planes, and locating and shifting the highest-pressure point. After correcting the misalignment in the O-XY plane, the pressure scan is performed in the OXZ plane using the same region scan and displacement step. The pressure was recorded, and an inverse Fourier transform function was used to determine the holography measurement setup for the UA design depicted in Figure 3.

### 2.3. Phase Calibration Method

The ultrasonic actuator system employs phase modulation to manipulate micromachines. Consequently, the phase of each transducer, generated at the focus, must be matched with the phase calculated using the phase modulation algorithm. This paper proposes the phase reference calibration method (PRCM) to reduce position misalignment and phase discrepancies caused by acoustic interactions. The proposed calibration method ensures that the measured phase of each transducer at the desired location corresponds to the phase modulation value that has been calculated.

**Figure 4:**
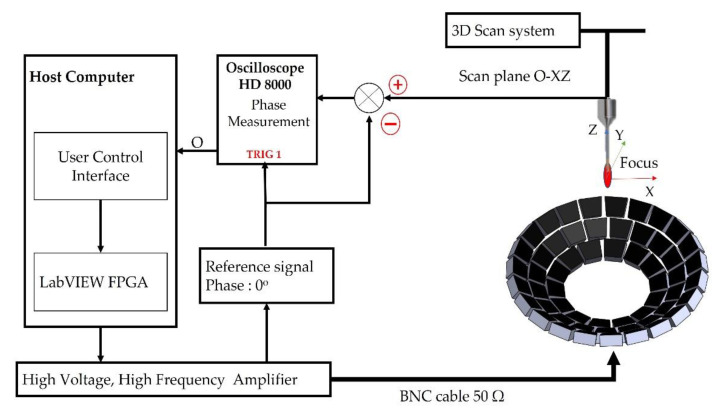
The control diagram to calibrate the UA using PRCM.

Figure 4 depicts the PRCM-based setup for calibration. The 56 identical transducers are categorized into three Z-axis circle layers. The calibration procedure can be carried out in a 300 mm × 300 mm × 300 mm container filled with de-ionized water. The phase calibration procedure using PRCM can be described as follows:

(1) Initialization: we set an initial phase offset value for each UT in the LabVIEW FPGA phase generation system, as well as a reference phase offset value for the zero-degree reference channel.
(2) Generating independent random phase values: we set three random phase values for each UT, then use TRIG 1 (HIGH edge of reference channel) to activate each UT with the reference channel.
(3) Applying the reference phase values: we compare the phase values of each UT with the phase values of the reference channel. In this step, three measurements are performed to determine the average measured phased value of the transducer, which is then compared to the reference channel.

(5) $$\delta_{\varphi i}=\sum_{j=1}^{j=3}\left(\varphi_{i}^{j}-\delta_{\varphi REF}\right)/3$$

$\delta_{\varphi i}$ denotes the measured phase of the transducer *i*, $\varphi_{i}^{j}$ denotes the phase offset value of transducer *i* in the *jth* measurement, and $\delta_{\varphi REF}$ denotes the phase of the reference channel.

(4) The phase modulation algorithm updates the calibrate phase as an offset value to the UT phase value. Next, we calculate the positive decimal part of the offset value.
  (6) $$\varphi i=\left[\begin{array}{ll} \frac{\delta_{\varphi i}}{360} & \;(\;1\;\geq \;\delta_{\varphi i}\geq \;0\;)\\ 1\;-\;\frac{\delta_{\varphi i}}{360} & \;(\;\delta_{\varphi i}\;\leq \;0\;) \end{array}\right.$$
(5) We set the updated phase offset value for each UT in the LabVIEW FPGA phase generation control program.

We use the LabVIEW FPGA control program’s single-cycle Timed Loop (SCTL) to quickly execute and optimize the code on the FPGA target PCIe 7852R. Although the system clockwork is set at 40 MHz, the phase offset for each UT is updated per single-cycle loop *t* ($t=\frac{1}{40\times 10^{6}}=25\;ns$). The system generates the control signal at 500 kHz and 3.3 V. As depicted in Figure 4, the control diagram for calibrating the UA using PRCM is configured as shown.

### 2.4. Manipulation of Micromachines Using Twin Trap

The proposed array will be applied in the manipulation of micromachines using a twin beam trapping method. In this method, the system creates the twin trap at the desired position to trap the targeted micromachine, and then the twin trap is regenerated at a new position to shift the micromachine to the new position. To generate an acoustic twin trap, the phase of each transducer was estimated as the sum of the focus phase and twin trap phase. The *π* radian difference (0.5 in phase offset value) was used to divide the array into two sides [29]. The phase of each transducer is given in Equation (7), as shown below:

(7) $$\varphi_{i}=\left[\begin{array}{l} 0+F_{i}\;with\;i=1:1:28\\ \pi +F_{i}\;with\;i=28:1:56 \end{array}\right.$$

*φ_i_* denotes the phase delay of transducer *i_,_* and *F_i_* denotes the phase of transducer *i* at the focus.

In addition, we mount two charge-coupled device (CCD) color cameras with 2.3 MP (Teledyne FLIR, Wilsonville, OR, USA) in the front and side of the water tank to track the target agent’s real-time position in the O-XZ plane and the O-YZ plane using LabVIEW Vision software (version 2017, Austin, TX, USA). The software will capture the background image without any targeted object. Then, this image will be compared with the real-time image, which captured the targeted object in 640 × 480 image resolution. Then, the position of the tracking object will be calculated in UA coordinates.

The compatibility between the commercial ultrasound imaging system and the proposed ultrasonic actuator will be verified through experiments in order to be used simultaneously in future clinical applications. In this experiment, we used the L12 ultrasound imaging probe that operates from 6.6 MHz to 12.3 MHz (Siemens Healthineers, Erlangen, Germany) to capture the target in the sub-mm scale. Then, the real-time position in the O-XZ plane of the targeted agent was calculated and compared with the desired position to show the position error.

## 3. Results and Discussion

### 3.1. Element-by-Element Acoustic Characteristic

For each transducer measurement, an electrical current of 0.02 A and 20 Vpp was recorded. Therefore, the electrical power transmitted to a single transducer was calculated to be 0.4 W, and that transmitted to all 56 transducers in the UA was 20 W. The acoustic pressure was calculated based on the waveform with an amplitude of 15 to 21 mV, corresponding to 19 to 27 kPa. At 20 Vpp, the total acoustic pressure generated by the UA is between 950 and 1350 kPa.

### 3.2. Holography Acoustic Characteristics

Before presenting the holography acoustic data of the UA, the misalignment angle between the UA coordinate and the hydrophone coordinate is measured. First, the OXY plane is scanned with the Z-axis = 0. The maximum pressure is located at the coordinates (0, 0, 0) in the hydrophone. Then, we scanned the second OXY plane with the Z-axis set to −40 mm. The maximum pressure was found at (0.2, 0, −40) as opposed to (0, 0, −40). The misalignment angle between the hydrophone coordinate and the UA coordinate can be calculated using this position as follows: $\delta_{\alpha }=\;\tan^{-1}(\frac{0.2}{40})\;=\;0.29{}^{\circ}$.

For further details, the manufacturer’s document should be consulted on misalignment angle calibration for the hydrophone. The angle δ_α_ = 0.29° has a negligible impact on the hydrophone signal’s relative amplitude. Therefore, to obtain the actual value of the pressure, we calibrate the hydrophone’s alignment angle to the true value.

**Figure 5:**
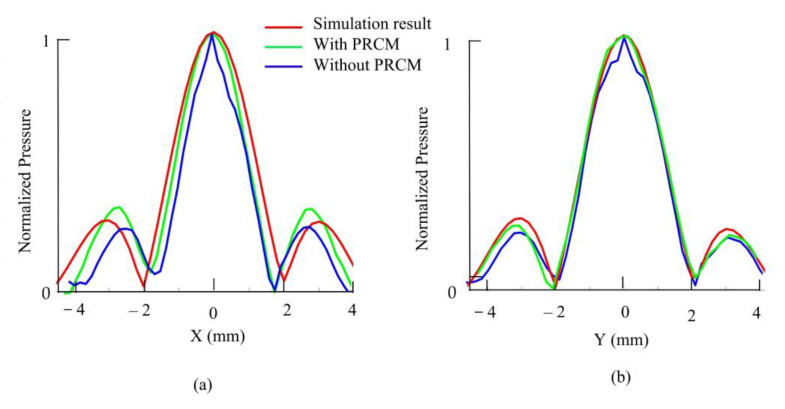
The comparison of beam size in cross plane at focus: (**a**) the O-XY plane view; (**b**) the O-XZ plane view.

The acoustic radiation field was simulated using COMSOL Multiphysics (COMSOL lnc., Burlington, MA, USA) at 500 kHz to describe the UA’s holography acoustic properties. We implemented a single-frequency, far-field piston model with acceleration control to simulate the vibration of a single transducer. All piston models exhibited sinusoidal control functions. We exported the pressure radiation values and used three plot functions in MATLAB (Ver. 2018, The MathWorks, Inc., Natick, MA, United States) for graphing them. We then conduct a 3D scan of the single beam generation and twin trap beam. Then, PRCM is utilized to reduce the position misalignment and phase differences caused by acoustic interactions. In the single beam generation, all UA transducers were driven with the same phase offset at 0° and bipolar voltage amplitude at 20 Vpp. As shown in Figure 5a for the cross plane at focus in the O-XY plane, the simulation result shows the main beam with a length from −2 mm to 2 mm, and a beam area of 3.18 mm^2^, with the peak pressure value at 0 mm. After applying the PRCM, the main beam has a length from −1.8 mm to 1.6 mm, and a beam area of 2.96 mm^2^, with the peak pressure value at 0 mm. Without applying the PRCM, the main beam has a length from −1.6 mm to 1.6 mm, and a beam area of 2.63 mm^2^, with the peak pressure value at 0 mm.

As shown in Figure 5b for the cross plane at focus in the O-XZ plane, the simulation result shows that the main beam has a length from −2 mm to 2 mm, and the beam area is 3.18 mm^2^, with the peak pressure value at 0 mm. With the PRCM, the main beam has a length from −2 mm to 2.2 mm, and the beam area is 2.90 mm^2^, with the peak pressure value at 0 mm. Without applying the PRCM, the main beam has a length from −1.8 mm to 2.2 mm, and the beam area is 2.76 mm^2^, with the peak pressure value at 0.2 mm. Thus, applying the PRCM increases the beam shape by 10.34% in the O-XY plane and 4.4% in the O-XZ plane. The peak pressure was regenerated at the designated focus point at (0, 0, 0) and the pressure amplitude around the focus area changes smoothly in the simulation result. This result will make the twin trap more stable.

**Figure 6:**
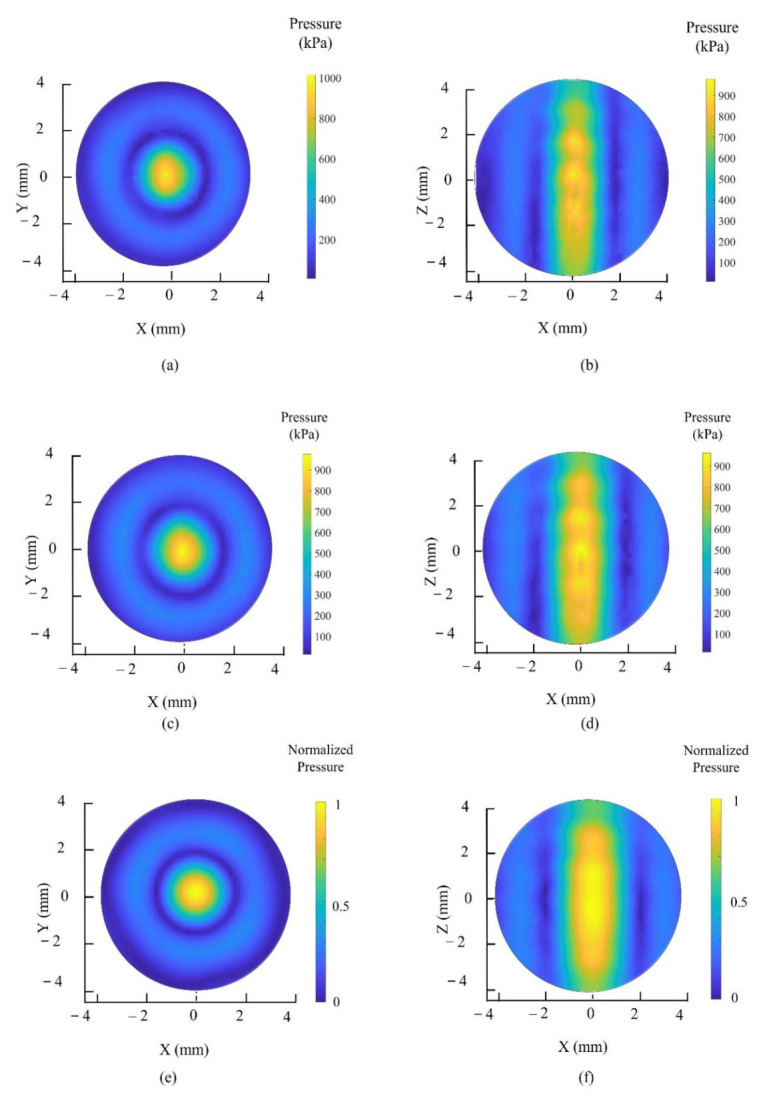
The 3D scanned pressure field for a single beam: (**a**) the scanned pressure in the O-XY plane; (**b**) the scanned pressure in the O-XZ plane; (**c**) the scanned pressure in the O-XY plane with PRCM; (**d**) the scanned pressure in the O-XZ plane with PRCM; (**e**) the simulation pressure in the O-XY plane with 200 μm of each data point; (**f**) the simulation pressure in the O-XZ plane with 200 μm of each data point.

Figure 6 depicts the scanned pressure field for a single beam in the O-XY and O-Z planes in the presence and absence of PRCM, respectively. The simulation results depicted in Figure 6e,f show the pressure radiation field map for a homogeneous area of focus. As shown in Figure 6a,b, prior to the application of PRCM, the maximum acoustic pressure was located at (0, 0, 0.2) with a pressure value of 1.06 MPa, as opposed to at the focus (0, 0, 0). As a result, the beam in the O-XZ plane is ununiform in one region. As depicted in Figure 6c,d, the maximum acoustic pressure was located at (0, 0, 0) with a pressure of 976 kPa after the application of PRCM. Compared to the result without PRCM, this result demonstrates that the proposed calibration method corrects the position of the focal point and makes the single beam more uniform around the foci area.

### 3.3. Generation of Twin Trap

**Figure 7:**
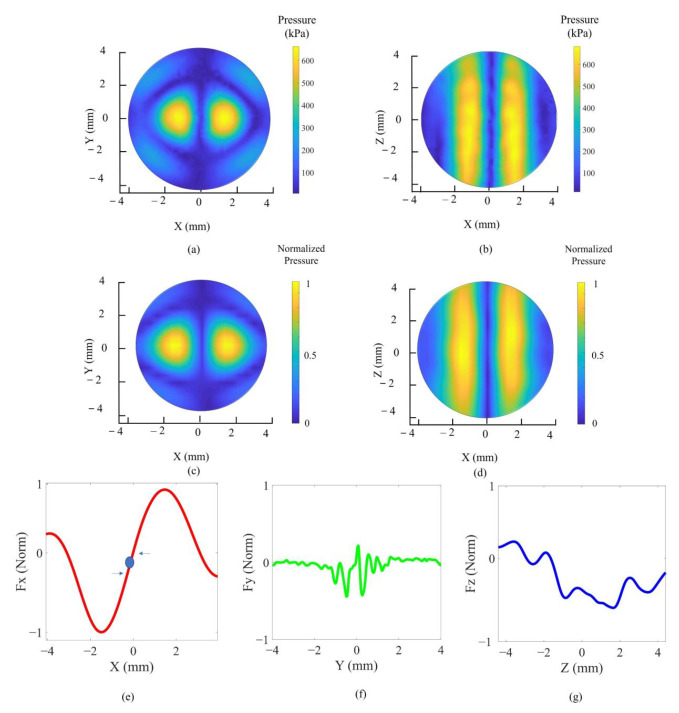
The 3D scanned pressure field for the generated twin trap with PRCM: (**a**) the O-XY plane view; (**b**) the O-XZ plane view; (**c**) the twin trap simulation result in the O-XY plane; (**d**) in the O-XZ plane; the simulation of acoustic radiation force normalization in the (**e**) X axis; (**f**) Y axis; (**g**) Z axis.

The UA was designed to utilize phase modulation to control the micromachine. Consequently, the twin trap was produced by applying PCRM. Figure 7 shows the 3D scanned pressure field for the twin trap with PRCM and the acoustic radiation force simulation.

In this demonstration, the transducer was driven independently with twin trap phase information ranging from −*π* to *π* radians at 20 Vpp bipolar voltage with the same amplitude. The trap point was located at (0, −0.2, 0.4), and the maximum acoustic pressure was measured as 700 kPa in the absence of PRCM. The scanned result after applying PRCM indicates that the trap point was corrected to (0, 0, 0) and that the maximum acoustic pressure was increased by 2% to 715 kPa. As shown in Figure 7c–e, the typical feature of the twin trap is that the lateral force of the X axis is the most significant force among the three axes.

### 3.4. Acoustic Manipulation of Micromachines

**Figure 8:**
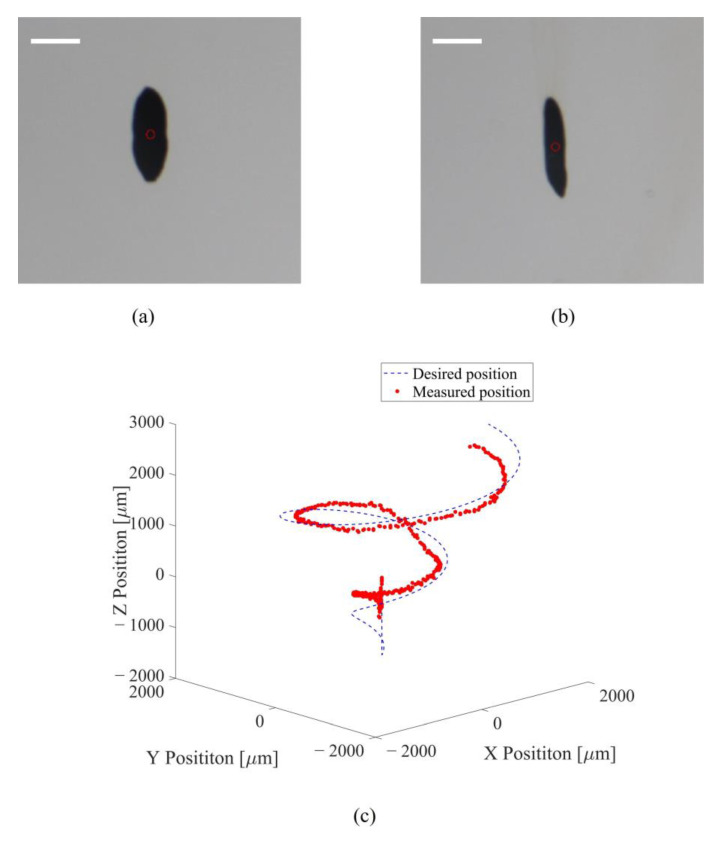
Demonstration of trapping and manipulating superparamagnetic iron oxide nanoparticles and polystyrene beads: (**a**) superparamagnetic iron oxide nanoparticles cluster was trapped in the O-XY plane view; (**b**) polystyrene beads cluster was trapped in the O-XY plane view; (**c**) manipulation of polystyrene beads cluster following helical trajectory (scale bar: 3 mm).

We demonstrated the ability to trap and manipulate target agents using the proposed acoustic actuator system. Superparamagnetic iron oxide nanoparticles (SPIONs) with a diameter of 100 nm and polystyrene beads (PBs) with a diameter of 100 μm were used as the target agents. Figure 8 shows a demonstration of trapping and manipulating the SPIONs and PBs. In this demonstration, the target agent was trapped by using a twin trap, which was described in Section 3.3. We observed that SPIONs and PBs remain stable at the trapping position as a cluster with a size of 3 mm × 8 mm × 80 μm. Lastly, we demonstrated three-dimensional (3D) manipulation of the PBs cluster following the helical trajectory. The result shows that the cluster was trapped and manipulated following a preprogrammed trajectory with average position errors in the X direction of 274 ± 273 μm, in the Y direction of 119 ± 118 μm, and in the Z direction of 455 ± 424 μm, respectively. To reduce the position error, we will implement the closed-loop control with PID controller method in a future study. The optimization of the phase modulation method should also be studied.

### 3.5. Acoustic Manipulation under Ultrasound Imaging

**Figure 9:**
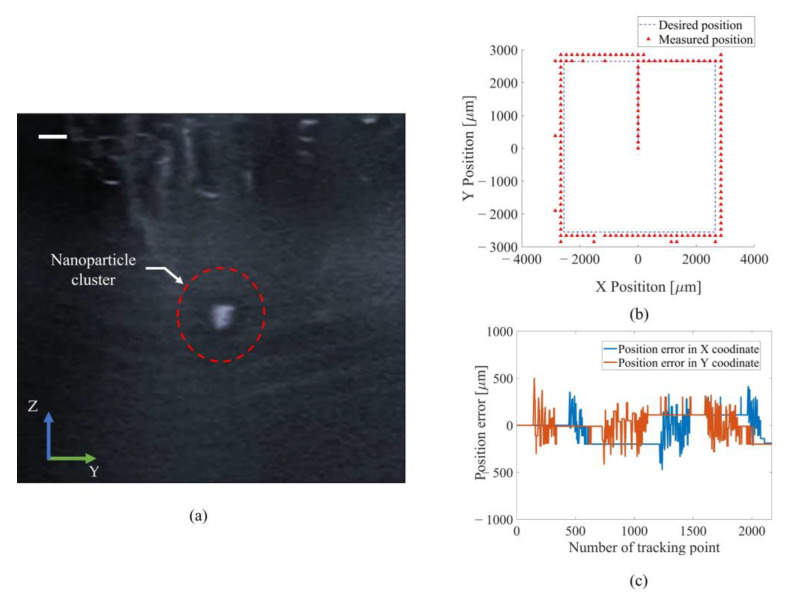
Demonstration of trapping and manipulating nanoparticles under ultrasound imaging: (**a**) the ultrasound imaging of nanoparticles; (**b**) the measured position of nanoparticles following preprogramed trajectory in the O-XY plane; (**c**) the position error in the O-XY plane (scale bar: 3 mm).

As shown in Figure 9, the SPIONs cluster could be imaged by the commercial ultrasound imaging system while minimizing any acoustic interference. Then, the SPIONs cluster was manipulated following a square trajectory with the dimensions 2.5 mm × 2.5 mm in the OYZ plane. The SPIONs cluster was successfully manipulated and followed the trajectory with a position error of less than 500 μm.

## 4. Conclusions

This article presents the design and fabrication of a 56-element acoustic actuator, which operates at a low frequency of 500 kHz, suitable focal distance of 75 mm, and low complex system for manipulating micromachines in a water medium. First, we simulated and characterized the acoustic fields using element-by-element and holography measurements. The element-by-element measurement is utilized to determine the characteristics of each transducer and the conversion efficiency of electric power to acoustic power. The holography measurement then reveals the UA’s total acoustic characteristics. Second, we proposed the phase reference calibration method (PRCM), which reduces position misalignment and phase discrepancies caused by acoustic interactions. Finally, we demonstrated the trapping and manipulation capabilities of micromachines in a water medium based on the acoustic twin trap, which was generated by the proposed acoustic actuator. The manipulation accuracy was analyzed by using real-time position information of the target agents that were tracked with the CCD camera. In addition, the compatibility between the commercial ultrasound imaging system and the proposed ultrasound actuator was verified. The SPIONs and PBs cluster remain stable at the trapping position and perform 3D motions following the helical trajectory, with average position errors of 274 ± 273 μm, 119 ± 118 μm, and 455 ± 424 μm on the X axis, Y axis, and Z axis, respectively. The proposed system and method would be applied to controlling micromachines as a targeted drug delivery system in the future.

## Figures and Tables

**Table 1:** Specification of the 56-channel model.

Description	Values	Unit
Number of elements	56	EA
Resonance frequency	500	kHz
System Input Voltage	60	Vpp
Radius of Curvature	120	mm
The distance from model surface to focal point	75	mm

## Data Availability

Not applicable.
